# Utilizing SS-OCT and 3D parametric modeling to enhance IOL position prediction in cataract patients with long axial length

**DOI:** 10.3389/fmed.2025.1604224

**Published:** 2025-06-27

**Authors:** Chuang Li, Bin Luo, Xiaoling Luo, Liying Zhang, Qiang Li, Wei Peng

**Affiliations:** Department of Ophthalmology, Shenzhen People’s Hospital, (The First Affiliated Hospital, Southern University of Science and Technology; The Second Clinical Medical College, Jinan University), Shenzhen, Guangdong, China

**Keywords:** effective lens position, lens equator position, intraocular lens, biometric parameter, 3D modeling

## Abstract

**Purpose:**

This study aimed to investigate the biological characteristics of the lens and explore the factors influencing the postoperative position of intraocular lens (IOL) using swept-source optical coherence tomography (SS-OCT) in cataract patients with long axial length (AL).

**Methods:**

This retrospective study enrolled 110 cataract patients who underwent uneventful cataract surgeries. The preoperative and postoperative biometric parameters were obtained using IOLMaster 700. The average lens density (ALD) was analyzed using Fiji ImageJ. The biological characteristics of cataracts were analyzed using three-dimension (3D) parametric modeling software, Pro/Engineer Wildfire 5.0.

**Results:**

The lens equator position (LEP) showed positive correlation with the preoperative anterior chamber depth (ACD), lens equatorial diameter (LED), and lens vaulting (LV). The postoperative position of the IOL showed positive correlation with the preoperative ACD and LEP (*r* = 0.432, *p* < 0.05; *r* = 0.657, *p* < 0.05). The LEP demonstrated a higher correlation with the postoperative position of the IOL than with the preoperative ACD (*p* < 0.05). The LEP displayed the highest correlation with the postoperative position of the IOL (*r* = 0.686, *p* < 0.05) in the subgroup of ultra-long eyes (AL ≥ 28.0 mm).

**Conclusion:**

The LEP, derived from the SS-OCT and 3D parametric modeling, was a better predictor of postoperative IOL position than the preoperative ACD. This methodology provides a robust basis for accurately predicting the effective lens position in cataract patients with long AL.

## 1 Introduction

The biological characteristics of the crystalline lens play an essential role in evaluating cataract morphology and calculating intraocular lens power (1). *In vivo* measurements of the human crystalline lens have primarily been conducted using slit-lamp, magnetic resonance imaging, Purkinje technique, Scheimpflug technique, and ultrasound biomicroscopy. Nevertheless, traditional imaging approaches are limited by subjectivity, low resolution, and inadequate penetration (2–5). The biological characteristics of isolated human eye bank lenses studied *in vitro* are often influenced by different balanced salt solutions, preservation time, and alterations in homeostasis (2).

Recently, the IOLMaster 700, which is based on swept-source optical coherence tomography (SS-OCT), has demonstrated excellent accuracy in intraocular lens (IOL) power calculation for cataract surgery, owing to its higher tissue resolution and faster scanning rate compared to other optical biometry methods. The IOLMaster 700 operates at a wavelength of 1,055 nm, with a scanning rate of 2,000/s, a depth of 44 mm, and a resolution of 22 μm. It can accurately capture the characteristics of both the anterior and posterior lens, free from the interference of refraction caused by the anterior and posterior surfaces of the cornea and lens, which can result from the distortion from the Scheimpflug camera (6).

Cataract phacoemulsification and IOL implantation are key approaches for improving vision. However, challenges in IOL power calculation persist, especially in cataract patients with long AL, despite the emergence of new predictive approaches. Accurate IOL power calculation prediction, based on the biological characteristics of the crystalline lens and the estimation of the effective lens position (ELP), plays an essential role in eyes with long AL. Olsen confirmed that the C constant is valuable for accurately predicting IOL position using optical biometry, based on the position and dimension of the preoperative crystalline lens, specifically preoperative ACD and lens thickness (7). Shammas proposed using the ante-nucleus distance to improve the estimation of ELP and enhance the accuracy of IOL power calculation (8).

Pro/Engineer Wildfire 5.0 is a computer-aided design and drafting (CADD) software for three-dimensional (3D) modeling, developed by Parametric Technology Corporation (PTC, USA). It utilizes surface parametrization, which is based on distance, surface normals, and curvature, for applications such as vascular analysis in animal models, joint replacement modeling, and oral implants (9–11). A previous study employed specialized equipment with custom-developed segmentation 3D approaches to improve refractive and visual outcomes (12). To the best of the author’s knowledge, the biological characteristics of the crystalline lens in cataract patients with long AL, measured using 3D modeling and Fiji ImageJ software, include lens volume (VOL), distance from the center of the anterior cornea to the lens equator (LEP), lens equatorial diameter (LED), lens vaulting (LV), radius of curvature of the best fitting sphere of anterior lens surface (RAL), radius of curvature of the best fitting sphere of posterior lens surface (RPL), frontier lens surface area (FLSA), posterior lens surface area (PLSA), total lens surface area (TLSA), and average lens density (ALD). These parameters, based on SS-OCT, have not been previously reported and require further exploration.

This article aims to study the biological features of cataracts for VOL, LEP, LED, LV RAL, RPL, FLSA, PLSA, TLSA, and LSA in long eyes using 3D modeling software Pro/Engineer and Fiji ImageJ based on the IOLMaster 700 (SS-OCT) in order to improve the predicted accuracy of ELP in cataract surgery and provide relevant rationale in the future.

## 2 Methods

### 2.1 Patients

This retrospective consecutive case-series study was performed at Shenzhen People’s Hospital, the First Affiliated Hospital of the Southern University of Science and Technology. We enrolled 110 long AL cataract participants (110 eyes) who underwent cataract phacoemulsification surgery from September 2023 to August 2024 at Shenzhen People’s Hospital, Shenzhen, China. If both eyes were eligible, the first operated eye was included (13). Three types of hydrophobic acrylic IOL [SN60WF (Alcon), ZCB00 (Abbott Medical Optics), and ZA9003 (Abbott Medical Optics)] were used. The inclusion criteria were as follows: patients who underwent uneventful cataract surgery and had stable refraction results (≥ 1 month after surgery). Subgroup analyses were performed based on different ALs (24.5 mm ≤ AL < 28.0 mm and AL ≥ 28.0 mm). Patients with corneal opacity, ocular trauma, glaucoma, uveitis, retinal diseases, anisometropia, and postoperative best-corrected distance visual acuity worse than 20/40 were excluded according to the Update on Intraocular Lens Power Calculation Study Protocols by the American Academy of Ophthalmology (AAO) (13). This study conformed to the Declaration of Helsinki and was approved by the Institutional Review Board of Shenzhen People’s Hospital. The requirement for informed consent was waived because only the patients’ medical records were involved.

### 2.2 Lens density quantification by the SS-OCT

**Figure 1:**
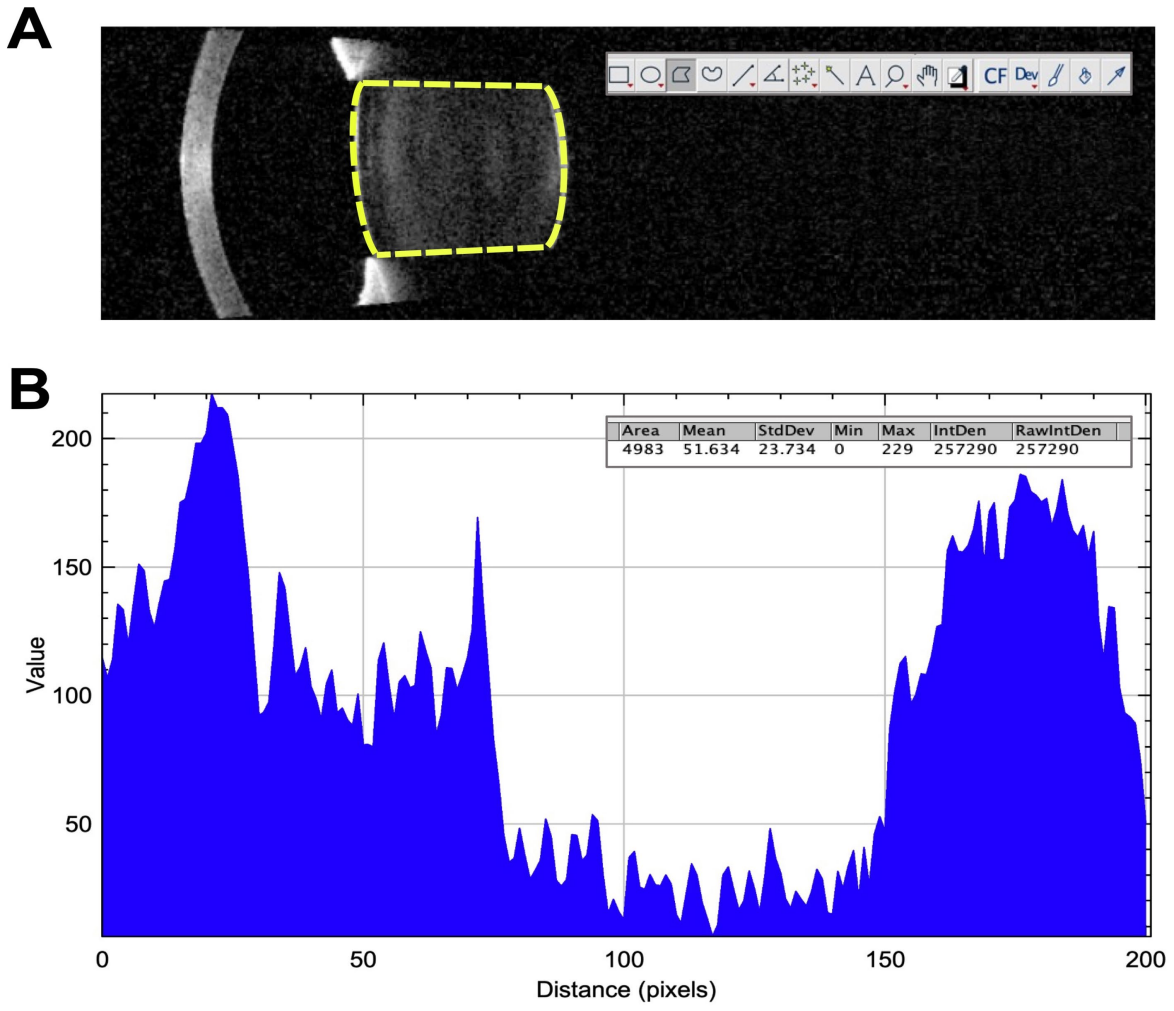
**(A)** The SS-OCT image of the lens exported to the ImageJ software and the lens density in the region of interest for measuring the cataract density in patients with long axial length. **(B)** The cataract density of the region of interest and the curve plot of the value of gray with Fiji ImageJ.

The latest SS-OCT (IOLMaster 700, Carl Zeiss, Germany) was used for lens imaging (14, 15). All patients in this study were measured preoperatively and postoperatively with the IOLMaster 700. It is capable of obtaining six successive series of high-resolution B-scan lens images along 6 meridians (0, 30, 60, 90, 120, and 150 degrees, respectively). Six B-scans were generated for each meridian three times. It uses a swept-source light at an emission wavelength of 1,055 nm, which obtains a scan depth of 44 mm and an axial resolution of 22 μm at a speed of 2,000 A-scans/s. The SS-OCT images of cataracts were analyzed according to a previously described method by Panthier et al. (16). All the SS-OCT images were analyzed using the Fiji ImageJ software (version 2.3.0/1.53f available at https://imagej.nih.gov/ij/; National Institutes of Health, Maryland, USA) for quantitative analysis with the undilated pupils. We manually delineated the anterior and posterior subcapsular boundaries and defined the region of interest (ROI) with a high level of precision using the Fiji Magic Wand tool of ImageJ (Figure 1). The ROI included the whole anterior and posterior capsule, including the lens cortex and nucleus. The density of cataracts was automatically analyzed by pixel intensity units, and higher pixel intensities indicated higher cataract densities (17). The ROI density was analyzed by the amplitude of 0 (pure black) to 255 (pure white) pixels. The Fiji ImageJ calculated the SS-OCT lens opacity as the ALD value derived from the corresponding readings along six meridians. The ALD and maximum lens density (MLD) were the average and maximum density (pixel intensity) of lens opacity in the region of interest after pharmacological mydriasis. The cataract density was analyzed on each of the six SS-OCT images. All the values obtained from lens density and 3D modeling were analyzed against the post-operative lens position to determine their correlation.

### 2.3 The 3-dimension (3D) parametric modeling analysis by the SS-OCT

**Figure 2:**
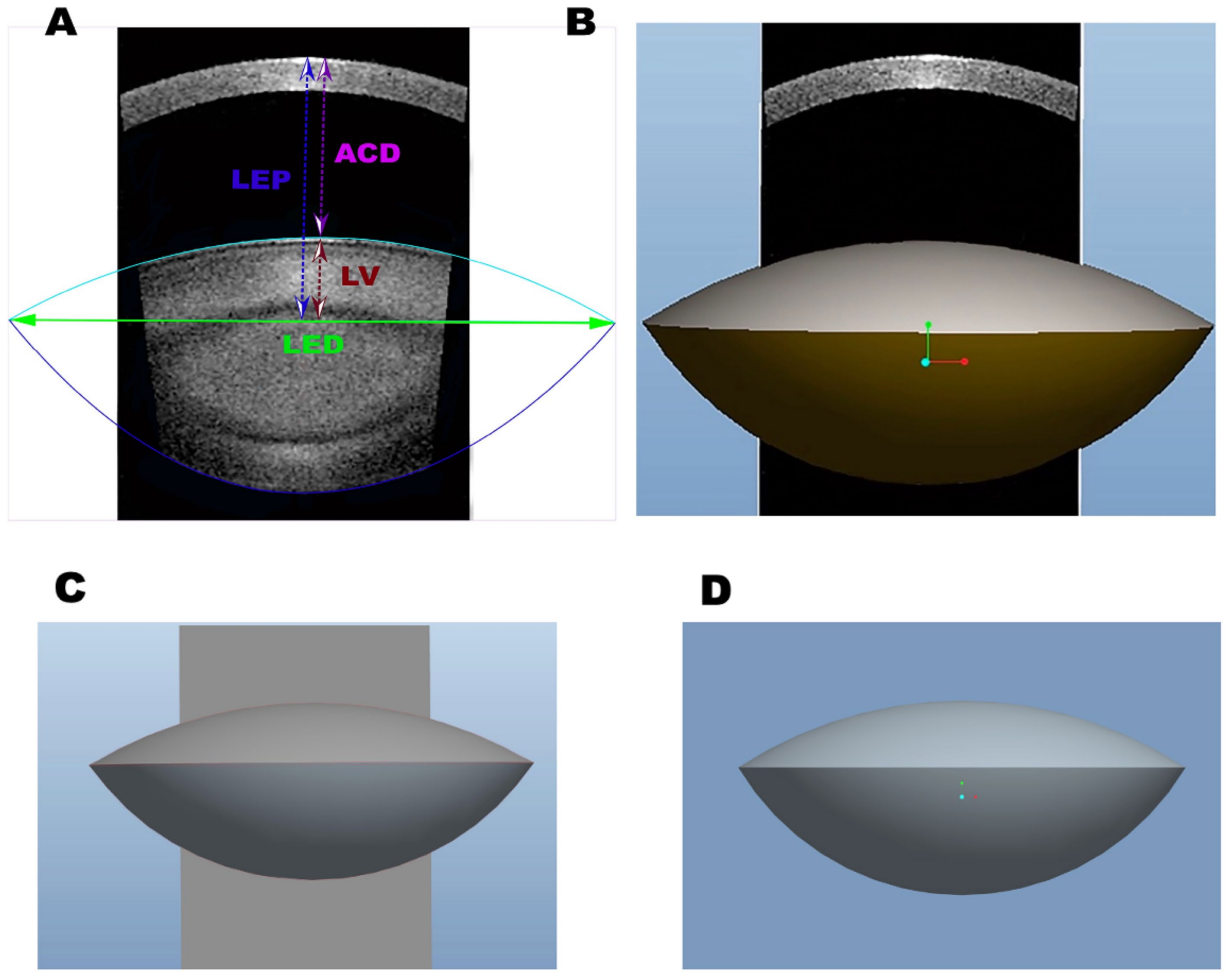
**(A)** The two-dimensional SS-OCT biological images of the lens and the characteristics of the lens, including equatorial diameter (LED), lens vaulting (LV), anterior chamber depth (ACD), and lens thickness (LT). **(B)** The three-dimensional biological features of the lens with SS-OCT. **(C)** The three-dimensional biological images of the lens with Pro/Engineer Wildfire 5.0. **(D)** The three-dimensional reconstructed SS-OCT biological images of the lens in cataract patients with long axial length.

The SS-OCT images were analyzed using 3D parametric modeling software (Pro/Engineer Wildfire 5.0 PTC, Parametric Technology Corporation, USA) for quantitative analysis. Pro/Engineer not only rapidly enables design of individual parts but also records assembly relationships and generates completed 3D drawings, based on two-dimensional (2D) drawings, using a tool called “Sketcher.” The Sketcher tool enables us to create rough drawings in the section using lines, angles, or arcs and then input the precise dimensional values later. We built the 3D parametric modeling based on the 2D SS-OCT biological images of the lens in cataract patients with long AL (Figure 2). Key metrics (LEP, RAL/RPL ratios) were exported to the software. Three anchor points were manually marked per meridian for each surface (apex and two equatorial points). Anterior/posterior capsule boundaries were traced using Pro/Engineer’s *Sketcher* tool automatically. The anterior and posterior lens capsules were manually delineated by two independent observers using a consensus protocol (Figure 2). The region of interest (ROI) included the entire lens capsule (cortex and nucleus). To account for cataract heterogeneity, ROIs were defined by anatomical landmarks (lens equator, anterior/posterior peaks) rather than density thresholds, ensuring consistency across patients.

**Figure 3:**
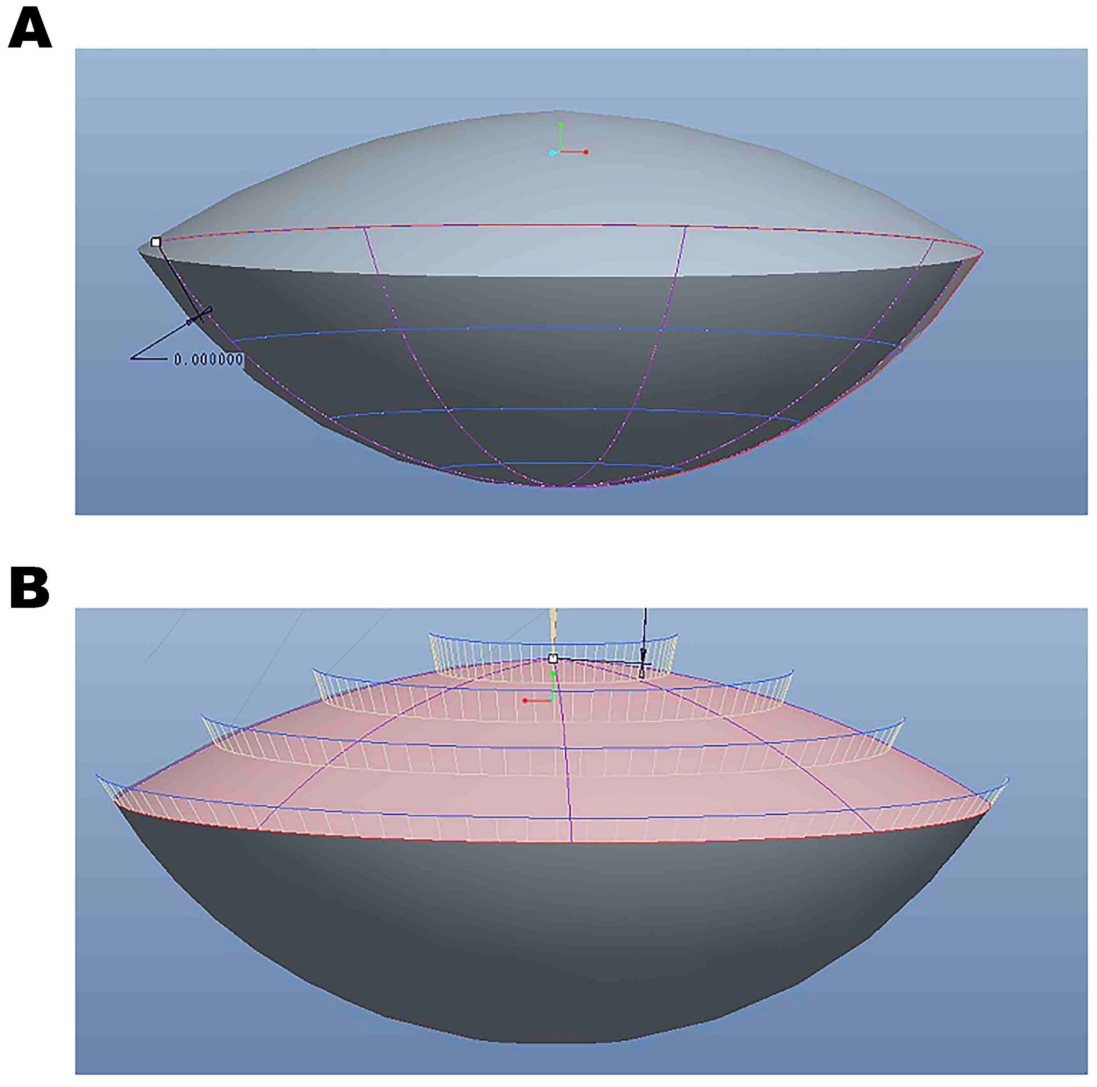
**(A)** The three-dimensional rendered images were edited using Pro/Engineer Wildfire 5.0. **(B)** The three-dimensional reconstruction data analysis based on SS-OCT images.

There are three basic Pro/Engineer design steps, including part creation, assembly creation, and drawing creation. It can access and edit dimensions and parametric associations at any stage of the workflow. The features start with a 2D section of SS-OCT lens images. When the section is defined, we can assign a 3D value to the SS-OCT lens images in order to make it a 3D shape. In addition, with the model analysis tools, we measure the assembly’s volume to determine its overall weight or center of gravity. The 3D parametric modeling software Pro/Engineer analyzed the biological characteristics of cataracts, including VOL, LED, LV, distance from the center of the anterior cornea to the lens equator position, intersection of the anterior and posterior lens obtained with SS-OCT (LEP), RAL, RPL, FLSA, PLSA, and lens surface area (LSA) (Figures 2, 3).

### 2.4 Statistical analysis

The analysis was carried out using SPSS (version 26.0, New York, USA). The Kolmogorov–Smirnov test was used to test the normal distribution. The Pearson correlation coefficients were calculated to assess the relationship between data that met the normal distribution. The intergroup variance was analyzed using a one-way analysis of variance (one-way ANOVA). Bonferroni correction was performed for multiple analyses. A *p*-value of < 0.05 was considered statistically significant.

## 3 Results

### 3.1 Baseline patient characteristics

The study included 110 eyes of 110 patients (mean age: 60.10 ± 11.45 years; male patients: 53). For bilateral cataract patients, only one eye was randomly selected for analysis. Preoperative ocular parameters were obtained using the IOLMaster 700 (Carl Zeiss, Germany), including axial length (AL), keratometry (flat, steep and mean), anterior chamber depth (ACD), lens thickness (LT), central corneal thickness (CCT), and cornea diameter (CD) (Table 1).

**Table 1:** Ocular biometric characteristics of participants (*n* = 110).

Variables	Mean ± SD	Med (median)
Age (years)	60.10 ± 11.45	62
Male patients	48.20%	NA
BCVA (logMAR)	0.49 ± 0.27	0.40
AL (mm)	27.97 ± 2.80	27.44
Flat K (D)	43.11 ± 1.61	43.36
Steep K (D)	44.21 ± 1.71	44.12
Mean K(D)	43.56 ± 1.50	43.36
IOL (D)	3.42 ± 0.38	3.53
ACD (mm)	4.40 ± 0.47	4.51
LT (mm)	546.43 ± 60.74	543.27
CCT (mm)	11.95 ± 0.49	11.86
CD (mm)	11.96 ± 0.48	12.00

BCVA, best-corrected visual acuity; logMAR, logarithm of the minimum angle of resolution; ACD, anterior chamber depth; AL, axial length; Flat K, flat keratometry; Steep K, steep keratometry; Mean K, mean keratometry; CCT, central corneal thickness; CD, cornea diameter; D, diopter; LT, lens thickness; *R*, correlation coefficient of bilateral biometric data.

### 3.2 The three-dimension (3D) parametric modeling analysis using the 3D parametric modeling software, Pro/Engineer Wildfire 5.0

The 3D parametric modeling analysis was performed using the parametric modeling software Pro/Engineer Wildfire 5.0. The LED was 10.25 ± 0.50 mm (range, 8.92–11.22), the VOL was 195.85 ± 37.26 mm^3^ (range, 123.56–311.33), the LEP was 4.97 ± 0.41 mm (range, 3.95–6.68), the RAL was 10.09 ± 0.32 mm (range, 9.35–11.74), the RPL was 6.03 ± 0.17 mm (range, 5.43–6.44), the FLSA was 89.67 ± 9.77 mm^3^ (range, 64.41–108.90), the PLSA was 109.81 ± 13.21 mm^3^ (range, 85.65–157.58), the TLSA was 199.48 ± 21.91 mm^3^ (range, 155.18–260.40), the LV was 1.47 ± 0.36 mm (range, 0.71–2.89), and the LB was 2.89 ± 0.41 mm (range, 2.09–4.37) (Table 2).

**Table 2:** Descriptive analysis including lens anatomy and position parameters using Pro/Engineer Wildfire 5.0 (*n* = 110).

Variables	Mean ± SD	Med (median)	Range (min, max)
LED (mm)	10.25 ± 0.50	10.25	8.92–11.22
LOV (mm^3^)	195.85 ± 37.26	193.46	123.56–311.33
LEP (mm)	4.97 ± 0.41	4.94	3.95–6.68
RAL (mm)	10.09 ± 0.32	10.09	9.35–11.74
RPL (mm)	6.03 ± 0.17	6.02	5.43–6.44
FLSA (mm^2^)	89.67 ± 9.77	89.57	64.41–108.90
PLSA (mm^2^)	109.81 ± 13.21	108.48	85.65–157.58
TLSA (mm^2^)	199.48 ± 21.91	198.24	155.18–260.40
LV (mm)	1.47 ± 0.36	1.51	0.71–2.89
LB (mm)	2.89 ± 0.41	2.88	2.09–4.37

LED, lens equatorial diameter; LOV, lens volume; LEP, distance from the center of the anterior cornea to the lens equator (intersection of the anterior and posterior lens obtained with SS-OCT); RAL, radius of curvature of the best fitting sphere of anterior lens surface; RPL, radius of curvature of the best fitting sphere of posterior lens surface; FLSA, frontier lens surface area; PLSA, posterior lens surface area; TLSA, total lens surface area; LV, lens vaulting; LB, distance between the posterior lens surface and the lens equator.

### 3.3 Lens density quantification by the SS-OCT

The Fiji ImageJ calculated the SS-OCT lens opacity as the ALD value derived from the corresponding readings along six meridians. The ALD and MLD were the average and maximum density (pixel intensity) of lens opacity. The ALD was 68.92 ± 14.90 pixel (range, 39.15–101.78), the MLD was 198.34 ± 34.90 pixel (range, 95.45–255.0), the LT/LED was 1.15 ± 0.14 (range, 0.83–1.50), and the LT/LV was 1.47 ± 0.36 (range, 0.71–2.89) (Table 3).

**Table 3:** Lens density quantification by the SS-OCT (*n* = 110).

Variables	Mean ± SD	Med (median)	Range (min, max)
ALD	68.92 ± 14.90	66.09	39.15–101.78
MLD	198.34 ± 34.90	195.56	95.45–255.0
LT/LED	1.15 ± 0.14	1.14	0.83–1.50
LT/LV	1.47 ± 0.36	1.51	0.71–2.89

ALD, average lens density in the ROI; MLD, maximum lens density, LED, lens equatorial diameter; LV, lens vaulting; LT, lens thickness.

### 3.4 The LEP exhibited a positive correlation with preoperative ACD, LED, LV, and LV/LT

The LEP exhibited a positive correlation with preoperative ACD, LED, LV, and LV/LT (*r* = 0.380; *r* = 0.306; *r* = 0.589; *r* = 0.630, *p* < 0.05). The LEP was not correlated with the AL, LT, ALD, MLD, VOL, and LT/LED (*r* = 0.137, *r* = 0.019, *r* = −0.068, *r* = −0.060, *r* = 0.141, and *r* = −0.106, *p* > 0.05) (Table 4).

**Table 4:** Correlation analyses of lens anatomy and position parameters from SS-OCT images with the LEP (*n* = 110).

Biometric parameters	LEP(mm)	
	*R*	*P-*value
AL (mm)	0.137	0.152
LT (mm)	0.019	0.930
ACD (mm)	0.380	0.000*
LED (mm)	0.306	0.030*
LV (mm)	0.589	0.000*
ALD (pixel)	−0.068	0.139
MLD (pixel)	−0.060	0.174
LOV (mm^3^)	0.141	0.435
LV/LT	0.630	0.000*
LT/LED	−0.106	0.270

AL, axial length; ACD, anterior chamber depth; LT, lens thickness; LOV, Lens volume; LED, Lens equatorial diameter; ALD, average lens density; LV, lens vaulting; MLD, maximum lens density; LEP, distance from the center of the anterior cornea to the lens equator position (intersection of the anterior and posterior lens obtained with SS-OCT).

^*^Statistically significant (*P* < 0.05).

### 3.5 The LEP exhibited a stronger correlation with the post-ACD than preoperative ACD

The postoperative position of the IOL (post-ACD) showed a positive correlation with both preoperative ACD and LEP (*r* = 0.432, *p* < 0.05; *r* = 0.657, *p* < 0.05). The LEP exhibited a stronger correlation with the postoperative position of the IOL than with preoperative ACD (*p* < 0.05) (Table 5).

**Table 5:** Correlation analyses of lens anatomy and position parameters from SS-OCT images (*n* = 110).

Biometric parameters	Post-ACD (mm)	
	*R*	*P-*value
LEP (mm)	0.657	0.001*
LT (mm)	−0.006	0.979
ACD (mm)	0.432	0.000*
LED (mm)	0.177	0.117
ALD (pixel)	−0.020	0.919
LOV (mm^3^)	0.101	0.371
LV (mm)	−0.078	0.492
RAL (mm)	0.011	0.922
RPL (mm)	0.089	0.453
FLSA (mm^2^)	0.129	0.255
PLSA (mm^2^)	0.162	0.152
TLSA (mm^2^)	0.151	0.182
LV/LT	0.195	0.084
LT/LED	−0.052	0.648

LEP, distance from center of the anterior cornea to the lens equator (intersection of the anterior and posterior lens obtained with SS-OCT); Post-ACD, postoperative intraocular (IOL) position; ACD, anterior chamber depth; LED, lens equatorial diameter; LT, lens thickness; LOV, lens volume; ALD, average lens density; RAL, radius of curvature of the best fitting sphere of anterior lens surface; RPL, radius of curvature of the best fitting sphere of posterior lens surface; FLSA, frontier lens surface area; PLSA, posterior lens surface area.

^*^Statistically significant (*P* < 0.05).

## 4 Discussion

Studies attributed 35.5% of non-systematic predictive errors to the ELP (18, 19). Many constants and biometric parameters were used to optimize the refractive outcomes in cataract patients with long AL (7, 8). Although several new formulas used more biometric variables or optical parameters, such as LT and CD, to predict the ELP (20), the accurate prediction of the ELP remains the major obstacle in formula calculation, particularly in cataract patients with long AL (21, 22). This study aimed to investigate whether the biological features of the crystalline lens in cataract patients, using 3D modeling and Fiji ImageJ software, which is based on SS-OCT, can be used to improve the predictive accuracy of IOL power calculation in cataract patients with long AL. Our results indicated that the LEP showed a higher correlation with the postoperative position of the IOL than with preoperative ACD, making it a better predictor of postoperative IOL position. This suggests that the LEP can influence the postoperative position of the IOL and can contribute to improved accuracy in IOL power calculation for cataract patients with long AL.

It is known that the biological features of the crystalline lens play an essential role in the intraocular lens power calculation. In the present study, we utilized the 3D parametric modeling and lens quantitative method to investigate the biological features of the crystalline lens with cataract. The latest commercial SS-OCT was recently used for lens imaging and density measurement, which uses an infrared swept-source light with the emission wavelength of 1,055 nm, which obtains a scan depth of 44 mm and an axial resolution of 22 μm at 2,000 A-scans/s (15, 23, 24).

The findings in this study indicated that the LED was 10.25 ± 0.50 mm (range, 8.92–11.22) and the VOL was 195.85 ± 37.26 mm^3^ (range, 123.56–311.33). Previous studies showed that the LED was 10.7 ± 0.50 mm (range, 7.5–11.9 mm) and VOL was 230.4 ± 31 mm^3^ (range, 119.9–312.4) mm using an SD-OCT femtosecond laser imaging system (Catalys Laser System; Johnson & Johnson Vision, Santa Ana, California, USA) (25), which were similar to our present results. Moreover, the current study showed that the RAL was 10.09 ± 0.32 mm (range, 9.35–11.74) and the RPL was 6.03 ± 0.17 mm (range, 5.43–6.44) (26). Previous studies have reported that the RAL and RPL were 10.21 mm and 5.75 mm, respectively, which are very similar to our results obtained using the 3D parametric modeling software Pro/Engineer Wildfire 5.0. Moreover, this study revealed that the ALD was 68.92 ± 14.90 pixels (range, 39.15–101.78), the MLD was 198.34 ± 34.90 pixels (range, 95.45–255.0), and the LEP was 4.97 ± 0.41 mm (range, 3.95–6.68). However, the LEP was not correlated with the AL, LT, ALD, MLD, VOL, and LT/LED (*r* = 0.137, *r* = 0.019, *r* = −0.068, *r* = −0.060, *r* = 0.141, and *r* = −0.106, *p* > 0.05).

Olsen and Shammas used recently developed constants and biometric parameters, such as the C constant and ante-nucleus distance, to predict the ELP accurately (7, 8). The challenges in IOL power calculation persist, although new approaches in long eyes continue to emerge. Yoo et al. showed that LEP is a promising parameter of preoperative crystalline lens geometry that can serve as an accurate predictor of postoperative IOL position using a femtosecond laser-assisted cataract surgery system (Catalys: AMO, North Chicago, IL) (27). Nevertheless, the requirements for the patient to be in a supine position and the significantly increased surgical costs make its widespread application difficult. Meanwhile, the Catalys integrates a spectral-domain intraoperative OCT with a short wavelength (1,000 nm) and axial resolution (30 μm) compared to the SS-OCT IOLMaster 700 (>1,000 nm) and 22 μm, respectively (28). Furthermore, Horiguchi et al. indicated that the duration of the scan was different (7 s for the Catalys intraoperative OCT versus 1.2 s for the SS-OCT IOLMaster 700) (29).

Objective and standardized quantification of the lens can significantly contribute to the accurate evaluation of the biological features of the crystalline lens in cataract patients. Consequently, Fiji ImageJ software was used to quantify the density of the lens with the SS-OCT images in our study. Furthermore, in this study, the Pro/Engineer Wildfire 5.0 was also applied to investigate the 3D biological features of the crystalline lens in cataract patients. It uses surface parametrization, which is based on distance, surface normal, and curvature, to define the 3D biological model and implants (9–11). The findings in this study identified that the postoperative position of the IOL (post-ACD) showed a positive correlation with the preoperative ACD and LEP (*r* = 0.432, *p* < 0.05; *r* = 0.657, *p* < 0.05) using the 3D parametric modeling software Pro/Engineer. The LEP showed a higher correlation with the postoperative position of the IOL than with preoperative ACD (*p* < 0.05). Furthermore, the current study found that the LEP and preoperative ACD showed a positive correlation with the postoperative position of the IOL in the subgroup analysis of different ALs (24.5 mm ≤ AL < 28.0 mm and AL ≥ 28.0 mm). In particular, the LEP showed the highest correlation with the postoperative position of the IOL (*r* = 0.686, *p* < 0.05) in the subgroup of ultra-long eyes (AL ≥ 28.0 mm) (Table 6). Compared to traditional ELP predictors, LEP demonstrates several biomechanical advantages. Unlike the C constant and ante-nucleus distance, which rely solely on anterior segment measurements, LEP incorporates both anterior and posterior lens curvature data from SS-OCT, which may provide more complete anatomical characterization.

**Table 6:** Correlation analyses of lens anatomy and position parameters with the LEP in the subgroup analysis (*n* = 110).

Biometric parameters	Post-ACD (mm)		Post-ACD (mm)	
	(24.5 mm ≤ AL < 28 mm)		(AL > 28 mm)	
	*R*	*P-*value	*R*	*P*-value
LEP (mm)	0.621	0.004*	0.686	0.032*
LT (mm)	−0.079	0.564	−0.249	0.230
ACD (mm)	0.385	0.004*	0.526	0.007*
LED (mm)	0.212	0.120	0.087	0.681
ALD (pixel)	−0.092	0.169	0.203	0.341
VOL (mm^3^)	0.162	0.237	−0.129	0.539
LV (mm)	−0.031	0.822	−0.351	0.086
RAL (mm)	0.069	0.616	0.222	0.286
RPL (mm)	0.014	0.918	0.306	0.137
FLSA (mm^2^)	0.190	0.164	−0.160	0.446
PLSA (mm^2^)	0.215	0.115	−0.007	0.975
TLSA (mm^2^)	0.210	0.124	−0.083	0.698
LV/LT	0.038	0.781	0.290	0.160
LT/LED	−0.022	0.874	−0.329	0.108

LEP, Distance from the center of the anterior cornea to the lens equator (intersection of the anterior and posterior lens obtained with SS-OCT); Post-ACD, postoperative intraocular (IOL) position; ACD, Anterior chamber depth; LED, Lens equatorial diameter; LT, Lens thickness; LOV, Lens volume; ALD, average lens density; RAL, Radius of curvature of the best fitting sphere of anterior lens surface; RPL, Radius of curvature of the best fitting sphere of posterior lens surface; FLSA, Frontier lens surface area; PLSA, Posterior lens surface area.

The main strength of our present research lies in the fact that optical measurements using SS-OCT (IOLMaster 700) were performed with undilated pupils, as compared to the study by Yoo YS et al., which used a spectral-domain intraoperative OCT that required pupil dilation. It is worth noting that the use of mydriatics might have affected the results of ACD, especially in regard to the LT (29). In addition to that, another strength entails the objective and standardized quantification of lens density and 3D biometric parameters for VOL, LV, RAL, RPL, FLSA, PLSA, LSA, LV/LT, and LT/LED. However, the postoperative position of the IOL was not correlated with the LT, LED, ALD, VOL, LV, RAL, RPL, FLSA, PLSA, LSA, LV/LT, and LT/LED (*r* = −0.006, *r* = 0.177, *r* = −0.020, *r* = 0.101, *r* = −0.078, *r* = 0.011, *r* = 0.089, *r* = 0.129, *r* = 0.162, *r* = 0.151, *r* = 0.195 and *r* = −0.052, *p* > 0.05) (Table 5). The independence of LEP from AL, LT, and LT suggests its potential as a universal predictor of IOL position, especially in long eyes where conventional biometric parameters exhibit limited predictive accuracy. However, it necessitates prospective multicenter validation of refractive outcomes. For future clinical implementation, subsequent studies could modify existing IOL power calculation formulas by systematically substituting ACD parameters with adjusted values based on LEP. This adjustment would utilize the validated conversion factor derived from our 3D parametric modeling approach.

One limitation of this study is that biometric parameters were evaluated using the IOLMaster 700, which may limit the applicability of the findings to other instruments such as the Lenstar 900 (Haag-Streit AG, Köniz, Switzerland). Furthermore, the current workflow requires manual segmentation of SS-OCT images. Future studies should focus on developing machine learning-based automated segmentation algorithms to eliminate observer variability. Another limitation is that, although the study shows a correlation between the LEP and post-operative lens position, there is no analysis or method for using the LEP to predict whether the lens position leads to more accurate clinical refractive outcomes. This limitation emphasizes the critical need for future well-powered, multicenter prospective studies implementing standardized refractive assessment protocols in cataract patients with long AL. In addition, we used the measured data of the central lens, which may introduce a potential systemic bias. Further studies are needed to establish a proper clinical setting with real measurements of the total anterior and posterior lens radii and ELP, as this could be more important in the future.

In conclusion, after evaluating the lens density and 3D biometric parameters via 3D modeling and Fiji ImageJ software, which is based on SS-OCT as compared with the postoperative position of the IOL, our study identified that the LEP was the better predictor of postoperative IOL position than the preoperative ACD and can be used to optimize the refractive status in cataract patients with long AL in the future. Notably, LEP exhibited the strongest correlation with postoperative IOL position in cases involving ultra-long eyes.

## Data Availability

The original contributions presented in the study are included in the article/supplementary material, further inquiries can be directed to the corresponding author.
